# *Smed-pou4-2* regulates mechanosensory neuron regeneration and function in planarians

**DOI:** 10.1101/2025.05.15.654132

**Published:** 2025-05-16

**Authors:** Ryan A. McCubbin, Mohammad A. Auwal, Shengzhou Wang, Sarai Alvarez Zepeda, Roman Sasik, Robert W. Zeller, Kelly G. Ross, Ricardo M. Zayas

**Affiliations:** 1 Department of Biology, San Diego State University, 5500 Campanile Dr., San Diego, CA 92182 USA; 2 Center for Computational Biology and Bioinformatics, University of California, San Diego, La Jolla, CA 92093 USA

**Keywords:** POU4 transcription factor, ciliated sensory neurons, mechanosensation, gene regulatory networks, terminal selector

## Abstract

POU4 homologs are involved in the development of sensory cell types across diverse species, including cnidarians, ascidians, and mammals. Whether these developmental regulators are reused during adult tissue maintenance and regeneration remains a fundamental question in regenerative biology. Here, we investigate the role of the *Schmidtea mediterranea* BRN3/POU4 homolog, *Smed-pou4-2* (*pou4-2*), in the regeneration of mechanosensory neurons. We find that *pou4-2* is regulated by the SoxB1 homolog, *soxB1-*2, and is expressed in a distinct population of ciliated sensory cells that detect water flow. Transcriptomic analysis of *pou4-2*-deficient planarians reveals enrichment for conserved genes associated with human auditory and vestibular function, suggesting that planarian rheosensory neurons share molecular features with mammalian inner ear hair cells. Expression of these conserved genes is abrogated by RNAi-mediated knockdown of *pou4-2*. To determine whether these transcriptional changes had functional consequences for mechanosensory neuron identity or behavior, we next assessed the impact of *pou4-2* knockdown on sensory function. *pou4-2* RNAi results in impaired mechanosensation in both uninjured and regenerating planarians. Together with the loss of terminal differentiation markers in mechanosensory neurons, these findings identify *Smed-pou4-2* as a key regulator of mechanosensory neuron identity in planarians and support the idea that conserved sensory specification programs are redeployed during adult tissue regeneration.

## INTRODUCTION

**Figure 1:**
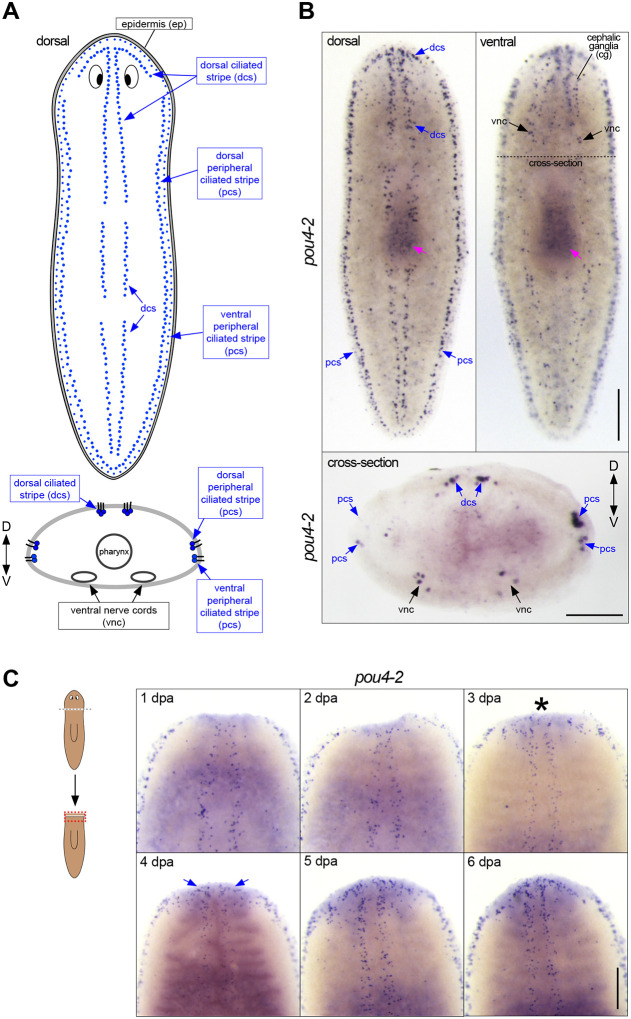
*Smed-pou4-2* is expressed in the ciliated stripes. (A) Planarian cartoon illustrates the dorsal and peripheral cell patterns implicated in detecting mechanosensory stimulation in *Schmidtea mediterranea*. (B) Whole-mount *in situ* hybridization to *pou4-2* revealed a stereotyped pattern depicted in (A), in the dorsal head tip, body periphery, dorsal ciliated stripe (dcs), and additional expression in the ventral nerve cords (vnc). The dashed line represents the cross-section plane shown in the bottom panel. Anterior is to the top. Scale bar = 200 μm. The cross-section image shows *pou4-2* expression in the dorsal ciliated stripe (dcs), dorsal and ventral peripheral stripes (pcs), and ventral nerve cords (vnc). Scale bar = 200 μm. (C) Expression analysis of *pou4-2*. The worms were amputated pre-pharyngeally, and regenerates were fixed at the designated time points. The asterisk highlights the first reappearance of expression within the blastema. The blue arrows at 4 dpa denote the appearance of the anterior dorsal ciliated stripe pattern. Anterior is to the top; dpa, days post-amputation. Scale bar = 200 μm; n ≥ 3 worms tested, with all samples displaying similar expression patterns.

Most animals, including mammals, have a limited capacity for neuronal regeneration. In contrast, organisms like fish and salamanders can effectively regenerate neurons, and some invertebrates are capable of dramatic whole-body regeneration. The freshwater planarian *Schmidtea mediterranea* is among a handful of research organisms capable of restoring virtually any lost or damaged tissue and can regenerate entire animals from small body fragments (Goldstein and Srivastava, 2022; Ivankovic et al., 2019). *S. mediterranea* possesses a population of adult pluripotent stem cells called neoblasts, which proliferate and differentiate to replace all missing tissues (Baguñà, 2012; Newmark and Sánchez Alvarado, 2002; Reddien, 2018). This stem cell population is postulated to include a heterogeneous pluripotent pool poised to acquire lineage-specific cell fates as needed (Raz et al., 2021). One of the extraordinary properties of planarians is the capacity for constant neuronal turnover and regeneration of neuronal cell types, many of which are conserved with vertebrates (Brown and Pearson, 2017; Ross et al., 2017). However, we know very little about the molecular basis of neurogenesis or the signals that regulate neuronal turnover (Lee, 2023). Previous studies found that *soxB1–2*, a mammalian Sox1/2/3 homolog, regulates the regeneration of ectodermal cell type subsets in planarians, including many uncharacterized sensory neurons (Ross et al., 2018). One prominent population of *soxB1-*2-regulated sensory cells, organized in a striking dorsal stripe pattern, functions in mechanosensation and is marked by *polycystic kidney disease-like* homologs (Ross et al., 2024; Ross et al., 2018) (Fig. 1A). Here, we sought to investigate mechanisms downstream of *soxB1–2* that are required to specify mechanosensory cells in the dorsal ciliated stripe.

POU transcription factor family genes play key roles in the development and function of many neuronal subtypes. To date, dozens of POU genes have been identified in vertebrates and invertebrates, and their roles in the differentiation and survival of diverse neuronal subtypes have been characterized (Leyva-Diaz et al., 2020). In many species, Brn3/POU4 transcription factors play important roles in specifying and maintaining the identities of various cell populations in the developing peripheral sensory nervous system. A notable example is *Nematostella vectensis NvPOU4*, which is required to maintain and differentiate cnidocytes, a population of mechanosensing cells exclusive to the phylum Cnidaria (Tourniere et al., 2020). The homologous role of *NvPOU4* in cnidarians suggests that the functional role of *pou4* is ancient and conserved across distantly related phyla. Additionally, *pou4* is part of a proneural regulatory cascade that produces epidermal sensory neurons in *Ciona intestinalis*; induction of ectopic *pou4* expression in the developing epidermis of *Ciona* larvae converts epidermal cells to sensory neurons, resulting in a striking hyper-ciliated phenotype (Chen et al., 2011).

In mice, *Pou4f3* is expressed exclusively in the inner ear sensory epithelia during embryonic development and is required for the survival of vestibular hair cells of the auditory system (Erkman et al., 1996; Xiang et al., 1997) - its targeted deletion results in impaired hearing and balance. Hair cells of the inner ear are crosslinked by stereocilia on their apical ends that function as mechanosensors, converting vibration-induced mechanical force into signals carried by auditory nerve fibers to the central nervous system (Goutman et al., 2015). Although a small number of hair cells differentiate in *Pou4f3*^−/−^ mice, their failure to form stereociliary bundles leads to apoptosis (Xiang et al., 1998). Thus, *Pou4* has conserved roles in the differentiation, maintenance, and survival of ciliated mechanosensory neurons. Unlike birds and fish, mammals lack the ability to regenerate hair cells after they are lost, resulting in permanent deafness (Edge and Chen, 2008). Recent studies show that POU4 can be used as a reprogramming co-factor to restore hair cells in mammals (Chen et al., 2021; Iyer et al., 2022). However, whether developmental regulators, like POU4, are reused during adult tissue maintenance and regeneration remains a fundamental question in regenerative biology (Seifert et al., 2023).

In *S. mediterranea*, a search for candidate planarian OCT4 homologs, a gatekeeper of pluripotency also known as POU5F1 in humans (Zeineddine et al., 2014), revealed six genes containing a POU-specific domain and a POU-homeodomain, and the two genes most similar to hPOU4F3 were named *Smed-pou4-1* and *Smed-pou4-2* (Önal et al., 2012b) (referred to as *pou4-1* and *pou4-2* hereon). *pou4-1* (also referred to as *pou4-like and pou4-like-1*) was identified as downstream of COE (Cowles et al., 2014), a transcription factor required for neurogenesis widely conserved across metazoans (Demilly et al., 2011), and is responsible for maintaining proper neuronal architecture in the cephalic ganglia as well as photoreceptor pigmentation (Cowles et al., 2014). More recently, our lab and others observed robust sensory defects in *pou4-2(RNAi)* planarians (Elliott, 2016; McCubbin, 2022; Wang, 2019). Under normal conditions, *S. mediterranea* worms display a stereotyped behavior by shortening their bodies in response to vibrations and water currents (rheosensation) across their dorsal side. We found that *pou4-2(RNAi)* planarians failed to react to this sensory input, indicating a significant role for *pou4-2* in mechanosensory neuron function (Elliott, 2016; Wang, 2019); however, its role in regenerative neurogenesis is not well understood. Here, we examined the function of *pou4-2* in mechanosensory neuron regeneration.

In this study, we mapped the expression of *pou4-2* and assessed its function through RNAi and RNA-seq. Loss of *pou4-2* expression coincides with loss of mechanosensation, which is not restored in regenerated *pou4-2(RNAi)* planarians. Analysis of the *pou4-2*^+^ cell gene expression profile uncovered that many genes regulated by Pou4-2 activity are necessary for proper mechanosensory neuron function and are homologs of human genes involved in hair cell function and auditory perception. In many organisms, the proneural *atonal* genes function in the same gene regulatory network as *pou4* (Leyva-Diaz et al., 2020). However, this relationship does not appear to be conserved in planarians. Our findings implicate *pou4-2* in a regulatory cascade that specifies distinct sensory neuron populations. This study demonstrates that *pou4-2* plays a key regulatory role in the differentiation, maintenance, and regeneration of ciliated mechanosensory neurons in planarians.

## RESULTS

### Smed-pou4-2 is expressed in planarian mechanosensory neurons

Our previous work demonstrated the key role of *soxB1–2* in sensory neuron differentiation and function of sensory neuron subclasses in the planarian *Schmidtea mediterranea* (Ross et al., 2018). A subset of *soxB1-*2-regulated genes are abundantly expressed in a discrete pattern called the dorsal and peripheral ciliated stripes (Figure 1A), which contain ciliated sensory neurons involved in detecting water flow (rheosensation) We took a candidate-based approach to gain a deeper mechanistic insight into how the sensory stripe cells are specified from a heterogeneous *soxB1–2*^+^ progenitor pool. POU4 genes are involved in the development of sensory organs detecting mechanical stimulation in divergent organisms (Manley and Ladher, 2008; Zhao et al., 2020). Thus, we investigated the expression and function of *S. mediterranea pou4* genes. The planarian genome encodes two POU4 homologs, *pou4-1* (also referred to as *pou4*-*like*) and *pou4-2* (Önal et al., 2012a). In previous work, we found that *pou4-1* (*pou4*-like) is expressed in the planarian CNS (Cowles et al., 2014). In contrast, analysis of *pou4-2* using whole-mount *in situ* hybridization (WISH) showed expression localized in the dorsal head tip and dorsal and peripheral ciliated stripes of intact planarians (Figure 1A–B), a stereotyped pattern common to rheosensory genes (Ross et al., 2018). In addition, *pou4-2* expression was detected in cells dispersed throughout the body in a subepidermal punctate pattern and in the cephalic ganglia and ventral nerve cords (Figure 1B). Because POU4 genes have been implicated as terminal selectors in widely divergent organisms (Leyva-Diaz et al., 2020), we examined the expression pattern in regeneration blastemas. During the first 24 hours of regeneration, *pou4-2* expression was absent from the blastema. We first detected clear expression on day 3 of regeneration (Figure 1C). The patterning of *pou4-2* expression in the blastema at day 3 was less organized and not confined to its normal spatial location, with *pou4-2*^+^ cells sparsely scattered throughout the regeneration blastema. During days 4 and 5, *pou4-2*^+^ cells began to repopulate the stereotypical stripe expression pattern, and by day 7, proper patterning was restored. In planarians, the regeneration blastema is populated by post-mitotic progenitors (Reddien, 2018). The delayed reestablishment of *pou4-2* expression suggests that it functions in the late stages of cell differentiation during regeneration.

**Figure 2:**
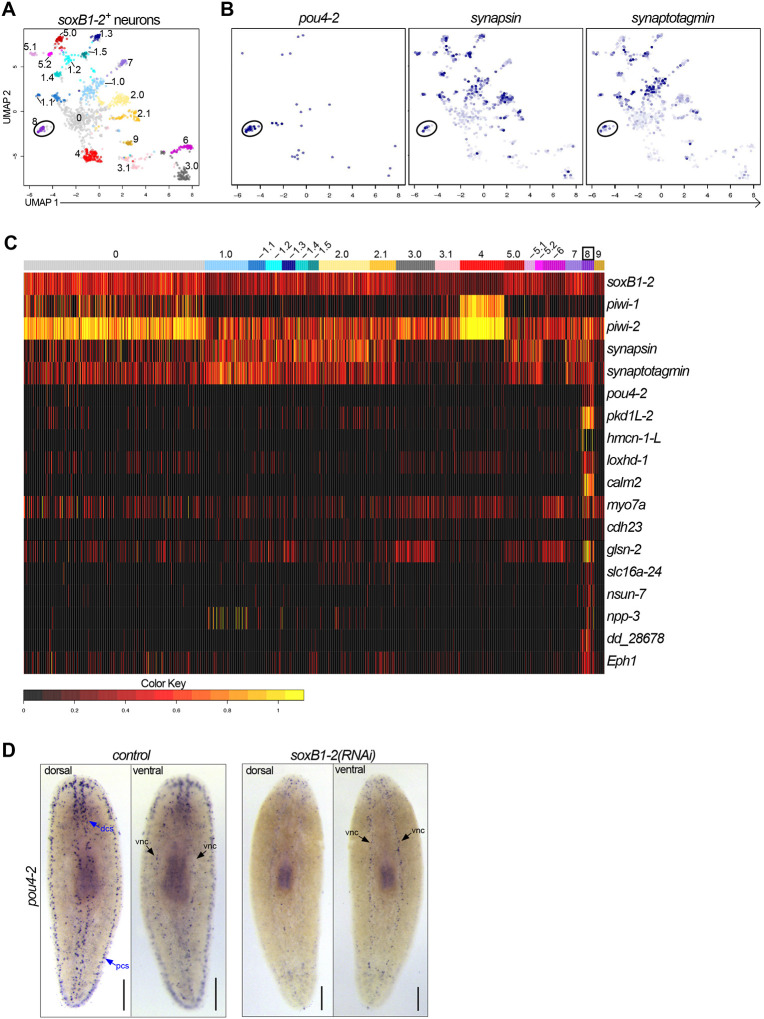
*Smed-pou4-2* is positively regulated by *soxB1–2*. (A) UMAP representation of *soxB1–2*^+^ neuronal subclusters (denoted by numbers) inferred from a scRNA-seq dataset. (B) *pou4-2*, *synapsin*, and *synaptogamin* are all enriched in the *soxB1–2*^+^ cluster 8. (C) Heatmap of genes examined in this study demonstrates their differential expression in *pou4-2*^+^ cells. (D) *soxB1–2* RNAi caused a reduction in sensory neuron-patterned *pou4-2* expression in the dorsal ciliated stripe (dcs) and the peripheral ciliated stripe (pcs) but did not affect ventral nerve cord expression (vnc). Anterior to the top. Scale bars = 200 μm; n ≥ 3 worms tested, with all samples displaying similar expression patterns.

*soxB1–2* is expressed in and regulates transcription in dorsal and peripheral ciliated stripe neurons as well as in other neural populations, and the epidermis of *S. mediterranea* (King et al., 2024; Ross et al., 2018). Therefore, we searched the existing scRNA-seq data from the entire body and brain (Fincher et al., 2018) to examine the potential relationship between *soxB1–2* and *pou4-2*. First, we extracted 1427 putative neuronal cells expressing *soxB1–2*. We resolved 19 distinct *soxB1–2*^+^ neuronal clusters (Supplementary File S1 and Figure 2A), of which cluster 8 was marked by *pou4-2*. The presence of *synapsin* and *synaptogamin* (neural markers) in cluster 8 indicated that these cells are neurons (Figure 2B). We combed through the dataset to identify genes that are differentially expressed in the *pou4-2*-enriched cluster (Figure 2C; Supplementary File S2); highly enriched genes included *pkd1L-*2 and *hmcn-1-L*, which are highly enriched in the planarian rheosensory organ (Ross et al., 2018). Because *pkd1L-*2 and *hmcn-1-L* expression requires *soxB1–2* activity, we hypothesized that *soxB1–2* regulates *pou4-2* expression. Thus, we treated planarians with *soxB1–2* or *pou4-2* dsRNA (the RNAi treatment scheme is depicted in Supplemental Figure 1A) and processed them for WISH. We observed a significant reduction in mechanosensory neuron-patterned *pou4-2* expression in *soxB1–2*(*RNAi*) planarians, whereas *pou4-2* expression in the central nervous system remained unaltered (Figure 2D). Conversely, *soxB1–2* expression was downregulated in mechanosensory neuron-patterned areas important for rheosensation (Supplementary Figure S2B). However, other areas enriched with *soxB1–2* expression, such as the auricles - anteriorly-positioned lateral flaps involved in chemotaxis (Almazan et al., 2021), the pharynx - an organ serving as the entrance and exit to the digestive system (Ishii, 1962), and the epidermis, were unaffected by *pou4-2* RNAi. We conclude that *soxB1–2* positively regulates *pou4-2* expression, specifically in mechanosensory neurons, but not in other cell types.

In many organisms, *pou4* and the proneural *atonal* genes are part of the same gene regulatory network (Leyva-Diaz et al., 2020). In *Ciona intestinalis*, *atonal* and *pou4* are part of a regulatory cascade downstream of Notch signaling that generates sensory neurons (Tang et al., 2013). In mice, *atoh1* is required for differentiation of multiple mechanosensory neuron types and stimulates expression of *pou4f3* to promote hair cell fate (Yu et al., 2021), and overexpression of *pou4f3* together with *atoh1* and *gfi1* in mouse embryonic stem cells can induce inner ear hair cell differentiation *in vitro* (Costa et al., 2015). There are three *atonal* homologs in the planarian genome, but none appear to operate in the same regulatory network as *pou4-2*. *atoh-1* is expressed in a discrete neuronal population in the cephalic ganglia, while *atoh8-1* and *atoh8-2* are expressed in stem cells in the mesenchyme, and all of them are involved in the regeneration of the nervous system (Cowles et al., 2013). *pou4-2* expression was unaffected after RNAi inhibition of all *atonal* genes in regenerated planarians (Supplementary Figure S2A); conversely, the expression of atonal genes was unaffected in *pou4-2* RNAi-treated regenerates (Supplementary Figure S2B). Thus, the functional relationship between *pou4* and *atonal* function observed in other animals does not appear to be conserved in planarians.

### *Smed-pou4-2* regulates genes involved in sensory neuron terminal fate

**Figure 3:**
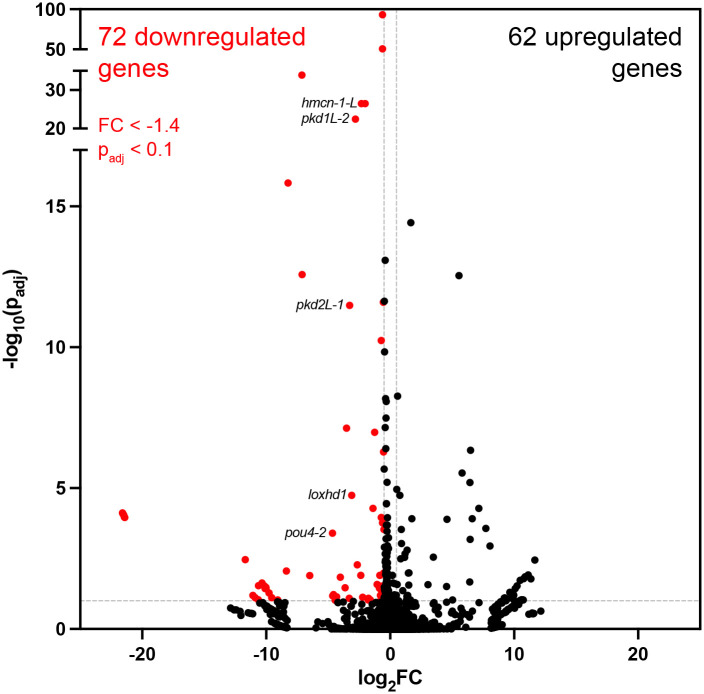
Identification of genes regulated by *Smed-pou4-2* using RNA-seq. Volcano plot of differentially expressed genes after *pou4-2* RNAi treatment in intact planarians.

Based on known roles of Pou4 genes, we hypothesized that *pou4-2* function might be required for cell differentiation and function. To test our hypothesis, we performed RNAi of *pou4-2* and pinpointed time points wherein the *pou4-2* transcripts were robustly downregulated and subsequently performed whole-animal RNA-seq on day 12 of the RNAi knockdown for *pou4-2(RNAi)* and control animals (see Methods). Analysis of the resulting data revealed downregulation of putative *pou4-2* target genes (Supplementary File S3). Because Pou4 genes are predicted to function as transcriptional activators, we focused further analyses on the downregulated gene set. *pou4-2* RNAi RNA-seq uncovered 72 significantly downregulated genes (Figure 3 and Supplementary File S3). GO analysis of the *pou4-*2-downregulated gene set revealed significant enrichment in ‘Mechanosensation’ (Supplementary File S4), including previously characterized genes we assessed to have roles in planarian mechanosensory modalities like vibration sensation and rheosensation, such as the polycystic kidney disease gene homologs *pkd1L-2* and *pkd2L-1* genes (Ross et al., 2024; Ross et al., 2018). This result is consistent with prior observations of the *pou4-2* RNAi phenotype in scRNA-seq studies and studies on the role of Notch signaling in planarians (Elliott, 2016). The discrete expression and *pou4-2* RNA-seq dataset motivated us to characterize the regulatory role of this transcription factor in planarian sensory neuron function and regeneration.

**Figure 4:**
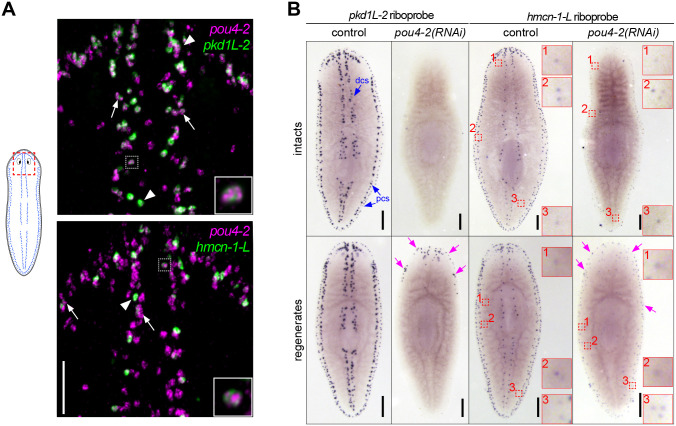
*pou4-2* is required for expression of *pkd1L-2* and *hmcn-1-L*. (A) Double-fluorescence in situ hybridization to visualize expression of *pou4-2* with *pkdL1–2* or *hmcn-1-L* in sensory neurons; 74.8% and 28.4% of *pou4-2*^+^ *cells were pkd1L-2*^+^ *and hmcn-1-L*^+^, respectively. (B) Planarians were fed twice per week for 4 weeks, amputated pre-pharyngeally one day after the last feeding, and allowed to regenerate for 10 days before fixation. Whole-mount *in situ* hybridization showed *pkd1L-2* and *hmcn-1-L* expression were drastically decreased in *pou4-2(RNAi)* worms. Blue arrows denote expression in the dorsal and peripheral ciliated stripes (dcs and pcs, respectively). Note that some *pkd1L-2* and *hmcn-1-L* expression was detectable in regenerates (red arrows). Regions denoted by a numbered dashed box indicate that *hmcn-1-L* expression was unaffected by *pou4-2* RNAi, which is shown in zoomed-in insets. Anterior is to the top. Scale bars = 200 μm; n ≥ 3 worms tested, with all samples displaying similar expression patterns.

The mechanosensory neurons in the rheosensory organ are distinguished by the expression of multiple sensory neural function genes and consist of at least two distinct populations, marked by the expression of terminal markers *polycystic kidney disease 1 like-2* (*pkd1L-*2) and *hemicentin-1-like* (*hmcn-1-L*) (Ross et al., 2018). The evolutionarily conserved PKD1L-2 is a cation channel pore component required for mechanosensation and cilia function (Patel, 2015); *pkd1L-2(RNAi)* planarians exhibit prominent rheosensory defects (Ross et al., 2024; Ross et al., 2018). On the other hand, *hmcn-1-L* is an extracellular matrix component involved in anchoring mechanosensory neurons to the epidermis (Vogel and Hedgecock, 2001); no detectable sensory defects are detected in *hmcn-1-L(RNAi)* planarians (Ross et al., 2018). Consistent with the scRNA-seq data, we found that a subset of *pou4-2*^+^ cells co-expressed *pkd1L-*2 or *hmcn-1-L*, representing two unique populations (Figure 4A). We consistently observed variable expression levels; some cells showed high expression of *pou4-2* and low expression of terminal markers (arrows in Figure 4A), while others showed lower expression of *pou4-2* but high expression of terminal markers (arrowheads in Figure 4A), and some had high expression of *pou4-2* and the terminal markers (white dashed box in Figure 4A). In addition, we observed that certain *pou4-2*^+^ cells were negative for *pkd1L-2* or *hmcn-1-L*, suggesting that they may represent undifferentiated progenitors. We reasoned that variable *pou4-2* expression might be due to *pou4-2* transcripts initially appearing in the progenitor cells to activate transcription of terminal markers and persisting at low levels in terminally differentiated cells.

To test whether this latter population of *pou4-2*^+^ cells constitutes sensory neuron progenitors, wild-type planarians were X-ray-treated with ~100 Gy, a dose reported to deplete early progenitors after 24 hours and late progenitors within 7 days (Eisenhoffer et al., 2008). We performed a WISH analysis every 12 hours post-irradiation and quantified the number of *pou4-2*^+^*/pkd1L-2*^−^*/hmcn-1-L*^−^ versus the number of *pou4-2*^+^ cells co-labeled with a single fluorophore mix of *pkd1L-2* and *hmcn-1-L* riboprobes (Supplementary Figure 3A). By 5.5 days post-irradiation (dpi), we noticed an obvious decrease in *pou4-2*^+^ cells negative for both terminal markers *pkd1L-2* and *hmcn-1-L* compared to the number of cells positive for *pou4-2* and either *pkd1L-2* or hmcn-1-L (Supplementary Figure 3B–C). WISH analysis was performed at various time points on irradiated animals to compare *pou4-2* expression with *prog-1* and *agat-1* expression, the latter function as early and late progenitors, respectively, in the epidermal differential lineage maturation trajectory (Eisenhoffer et al., 2008). The temporal downregulation of *pou4-2*^+^ cells is analogous to that of *agat-1*^+^ cells. In contrast, *piwi-1*^+^ neoblasts and *prog-1* early progenitors were almost entirely depleted by 2–3 dpi (Supplemental Figure 3D), which supports a role for *pou4-2*^+^ cells as late progenitors and the source of terminally differentiated mechanosensory neurons. Accordingly, we reasoned that variable *pou4-2* expression may be due to the transcripts initially appearing in the progenitor cells to activate transcription of terminal markers and persisting at low levels in terminally differentiated cells.

Next, we asked if *pou4-2* function is required to maintain and regenerate *pkd1L-2* and *hmcn-1-L* expression in the dorsal and peripheral ciliated stripes. We conducted WISH on intact and regenerated planarians treated with dsRNA over a time course (Supplemental Figure 1A). Like *pou4-2*, in control intact or regenerated planarians *pkd1L-2* and *hmcn-1-L* were expressed in the head tip, dorsal ciliated stripe, and dorsal and ventral peripheral stripes (controls in Figure 4B). However, in *pou4-2(RNAi)* animals, the expression of the marker genes was notably lost, especially in the uninjured worms. Interestingly, in the regenerates, minimal *pkd1L-2* and *hmcn-1-L* expression was observed in the regeneration blastema. We also detected lower levels of expression of *hmcn-1-L* at scattered locations near peripheral stripes that were unaffected by *pou4-2* RNAi (Figure 4B, dashed red boxes). It is possible that a longer *pou4-2* RNAi treatment might be necessary to ablate expression in those cells. We conclude that *pou4-2* is required for *pkd1L-2* and *hmcn-1-L* expression in the most prominent dorsal and peripheral ciliated stripe cells.

### *Smed-pou4-2* regulates genes involved in ciliated cell structure organization, cell adhesion, and nervous system development

**Figure 5:**
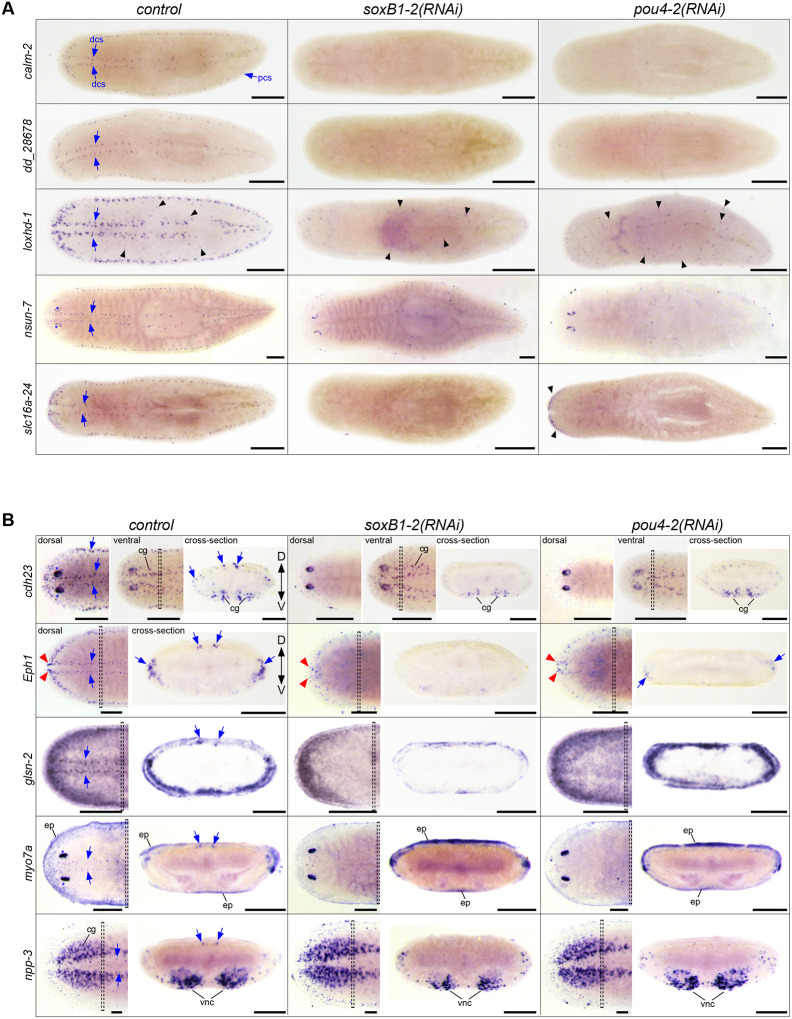
Expression analysis of genes co-expressed in *Smed*-*pou4-2*^+^ cells. (A) WISH images of genes predominantly expressed in the dorsal and peripheral ciliated stripes. WISH post-RNAi revealed reduced expression of mechanosensory neuron-patterned genes (labeled on the left) after *soxB1–2* and *pou4-2* RNAi (labeled on the top). *loxhd-1* was also expressed in a punctate pattern (black arrowheads) that appeared largely unaffected by *pou4-2* RNAi. The RNAi treatments did not affect *nsun-7* expression in the photoreceptors (blue asterisks). (B) In situ hybridization images from whole-mount and cross-sections of genes expressed in mechanosensory neurons and other cell types. Note reduced expression of mechanosensory neuron-patterned genes after *soxB1–2* and *pou4-2* RNAi. The red arrowheads highlight the tip cell expression unaffected by *soxB1–2* and *pou4-2* knockdown in *ephA4*. The insets show the corresponding cross-section of the worm. Anterior to the left. Blue arrows mark ciliated stripe cell regions. Dashed boxes denote cross-section regions. Abbreviations: cephalic ganglia (cg), dorsal ciliated stripe (dcs), dorsal and ventral peripheral stripes (pcs), epidermis (ep), ventral nerve cords (vnc). Scale bars = 200 μm for intact animals and 100 μm for cross sections; n ≥ 3 worms tested with all samples displaying similar expression patterns.

To further investigate the role of *pou4-2* in regulating the differentiation of mechanosensory neurons, we selected eight additional genes from either the RNA-seq *pou4-2* RNAi-downregulated genes or the *S. mediterranea* single-cell RNA-seq database (Figure 2–3; see Supplementary File S2 for the list of genes). Four of the genes, *cadherin-23*, *Smed-Eph1*, *lipoxygenase homology domain-1*, and *unconventional myosin VIIA*, are homologous to human proteins critical to the normal function of hair cells of the inner ear sensory epithelium, and their mutations lead to sensorineural hearing loss (described below). Using WISH and RNAi analysis, we determined their spatial expression patterns and whether they were downregulated by *pou4-2* and *soxB1–2* (Figure 5A–B). The expression patterns of *calmodulin-2* (*calm-2*), *lipoxygenase homology domain-1* (*loxhd-1*), and *dd_28678* were exclusively confined to the stereotypical mechanosensory neuron pattern in the head tip, body periphery, and dorsal ciliated stripe and completely depleted in *pou4-2(RNAi)* and *soxB1–2(RNAi)* planarians (Figure 5A). *pou4-2* RNAi-mediated loss of *calm2* expression is consistent with observations reported by King et al. (2024). Calmodulins are important for ion channel activity and signal transduction, and human *calm2* mutations are associated with delayed neurodevelopment and epilepsy (Crotti et al., 2013). *loxhd1* is predicted to encode a highly conserved stereociliary protein involved in hair cell function, and mutated human *LOXHD1* causes DFNB77, a form of progressive hearing loss (Grillet et al., 2009). *NOP2/Sun RNA methyltransferase family member 7* (*nsun-7*) was expressed in fewer cells in the mechanosensory neuron pattern overall, but an additional subset of *nsun-7*^+^ cells present in the optic cups was unaffected by *pou4-2* and *soxB1–2* inhibition, in contrast to clearly reduced *nsun-7* expression in sensory mechanosensory neuron-rich areas (Figure 5A). In humans, *nsun7* activity is required for proper flagella movement and sperm motility, and mutations result in male infertility (Khosronezhad et al., 2015). In control and *pou4-2(RNAi)* planarians, expression of the monocarboxylate transporter-encoding *solute carrier family 16 member 24* (*slc16a-24*) was detected in the auricles. Auricular *slc16a-24* expression was downregulated in *soxB1–2(RNAi)* but not *pou4-2(RNAi)* planarians, while expression in the mechanosensory neurons important for rheosensation was downregulated in both (Figure 5A).

Other genes we chose to analyze were not exclusively expressed in the ciliated stripes (Figure 5B). Expression of *cadherin-23* (*cdh23*) was highest in the photoreceptors, and low expression was detected in the epidermis. While *cdh23* expression was downregulated in the ciliated stripes, *cdh23*^+^ cells in the epidermis and photoreceptors were unchanged after *pou4-2* and *soxB1–2* inhibition. Human *CDH23* is expressed in the sensory epithelium of the inner ear, where it is involved in maintaining the stereocilium organization of hair cells required for sound perception and equilibrioception (Kazmierczak et al., 2007), and *CDH23* mutations are known to cause hereditary hearing loss (Woo et al., 2014). *Smed-Eph1* (*Eph1*), encoding an ephrin receptor homolog, was also among the differentially expressed genes in *soxB1–2*^+^*/pou4-2*^+^ neurons (Figure 2C). The role of Ephrin signaling in axon guidance is well-established and highly conserved; in mammals, the binding of ligand *efnb2* to receptor *EphA4* is critical to the differentiation and patterning of hair and support cells on the cochlear sensory epithelium (Defourny et al., 2019) and for targeting and innervating auditory projections to hair cells (Defourny et al., 2013). Consequently, *EphA4* mutations and failure to bind ligands are associated with sensorineural hearing loss in humans (Levy et al., 2018). While mechanosensory neuronal patterned expression of *Eph1* was downregulated after *pou4-2* and *soxB1–2* inhibition, low expression in the brain branches of the ventral cephalic ganglia persisted (Figure 4B). *Eph1* expression in the anterior-most region of the head tip was downregulated in *soxB1–2(RNAi)* but was unaffected in *pou4-2(RNAi)* planarians (Figure 4B). *gelsolin-2* (*glsn-2*) was highly expressed in the sensory neuron pattern and in the epidermis, where it appeared most abundantly expressed near the body periphery and weakly expressed in the medial dorsal surface (Figure 5A). Gelsolins’ roles in nervous system development (Mazur et al., 2016) and as modulators of ciliogenesis and cilium length are evolutionarily conserved (Kim et al., 2010). Sensory neuronal *glsn-2* expression was downregulated in *pou4-2(RNAi)* and *soxB1–2(RNAi)* planarians, and epidermal expression was also downregulated in *soxB1–2(RNAi)* planarians, potentially because of the role of *soxB1–2* in ciliated epidermal cell maintenance (Ross et al., 2018).

In the dorsal ciliated stripe, body periphery, and head tip, low expression of *unconventional myosin VIIA* (*myo7a*) was detected and depleted in *pou4-2(RNAi)* and *soxB1–2(RNAi)* planarians (Figure 5B). Additionally, *myo7a* was highly expressed in the photoreceptors, and this expression remained in *pou4-2(RNAi)* and *soxB1–2(RNAi)* planarians. The low expression of *myo7a* detected beneath the epidermis was also unaffected by *pou4-2* and *soxB1–2* inhibition. Human *myo7a* is important in stereocilium organization, differentiation, and signal transduction of inner ear hair cells (Jaijo et al., 2007). Defective *myo7a* and *cdh23*, to a lesser extent, cause Usher Syndrome Type 1B (USH1B), which is characterized by deafness and reduced vestibular function (Roux et al., 2006). *neuropeptide precursor-3* (*npp-3*) was highly expressed in the cephalic ganglia, ventral nerve cords, pharynx, and at lower levels in the parenchyma. A small subset of *npp-3*^+^ cells in the dorsal ciliated stripe was depleted in *pou4-2(RNAi)* and *soxB1–2(RNAi)* planarians (Figure 5B). Despite the presence of *pou4-2*^+^ cells in the ventral nerve cords (Figure 1B), *pou4-2* inhibition appeared not to affect npp-3 expression in that region (Figure 5B).

### Expression of *Smed-pou4-2* is required for mechanosensory neuron regeneration and function

**Figure 6:**
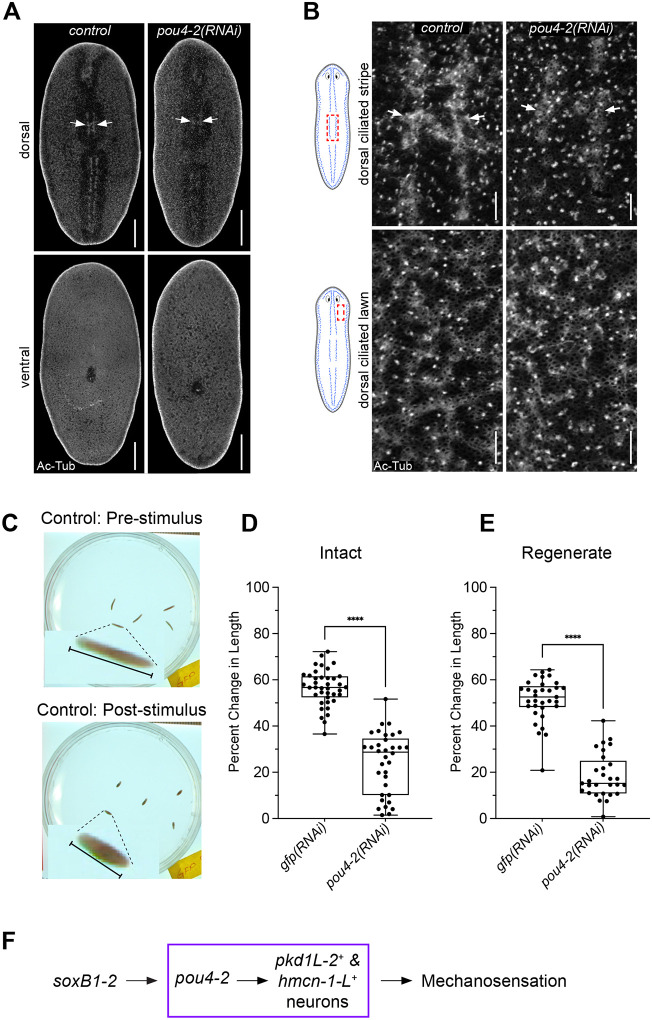
*pou4-2* expression is required for mechanosensory neuron regeneration and function. (A) Acetylated-tubulin staining revealed decreased cilia labeling along the dorsal ciliated stripe after *pou4-2* RNAi. Anterior is to the top. Scale bars = 200 μm, n = 4 worms stained for each of the control and experimental groups. (B) Higher magnification of acetylated tubulin staining of control and *pou4-2* RNAi animals. Scale bars = 25 μm. (C) Significant reductions in vibration sensation and rheosensation were observed in *pou4-2* RNAi worms. These reduced behaviors following stimulation were consistent in intact and regenerates. Data in C are represented as mean ± SD, and n > 25 worms for each experimental group. ****p < 0.0001, Student’s t-test.

Given that *pou4-2* expression is decreased in *soxB1–2*(*RNAi*) planarians (Figure 2D) and that *pou4-2(RNAi)* planarians have decreased expression of genes related to mechanosensory neuron and cilia function (Figure 4–5), we reasoned that subsets of *pou4-2*^+^ cells are terminally differentiated ciliated sensory neurons. Moreover, the requirement of *soxB1–2* for maintaining ciliated epidermal and sensory neuron populations supports the idea that *pou4-2* functions as a terminal selector in planarians. To assess the role of *pou4-2* in the dorsal and peripheral ciliated stripes, we first immunostained control and RNAi-treated planarians with anti-Acetylated-Tubulin to mark cilia. Compared to the controls, *pou4-2(RNAi)* planarians showed decreased cilia labeling along the dorsal ciliated stripe, while ciliated lawns on the dorsal and ventral surfaces remained unchanged (Figure 6A–B). To investigate the role of *pou4-2* in mechanosensory function, we used a semi-automated behavioral assay to evaluate the mechanosensory response to vibration stimulation (Ross et al., 2024) (Figure 6C). Knocking down *pou4-2* caused a significant attenuation of mechanosensory responses compared to control animals (Figure 6D). Moreover, the vibration stimuli produced significantly reduced responses from the regenerated RNAi animals (Figure 6E). Thus, we conclude *pou4-2* is downstream of *soxB1–2* and is necessary for maintaining and regenerating ciliated mechanosensory neurons in the rheosensory organ (Figure 6F).

## DISCUSSION

### *Smed-pou4-2* plays a key role in the regulation of sensory system differentiation

The interplay between lineage-specifying transcription factors and their respective gene regulatory networks coordinates precise developmental processes and stem cell fate decisions. POU4 transcription factors are conserved terminal selectors of sensory neuron fate, but the role of *Pou4* has not been extensively characterized in regeneration. This study’s objective was to elucidate the role of *Smed-pou4-2* (*pou4-2*) in planarian sensory neuron regeneration and to identify gene regulatory network components responsible for maintaining mechanosensory neuron function. *pou4-2*^+^ cells include ciliated mechanosensory neurons that allow planarians to detect water currents and vibrations (rheosensation), a function similar to *POU4F3* in hair cells of the mammalian inner ear sensory epithelium responsible for auditory perception and equilibrioception. We showed that *pou4-2* regulates the expression of genes whose homologs are involved in stereocilium organization, cell adhesion, and nervous system development in other organisms, and their human homologs are implicated in sensorineural hearing loss. These findings shed light on the molecular mechanisms underlying planarian regeneration and provide insight into the conserved role of POU4 transcription factors in sensory neuron development across divergent species.

Analysis of *pou4-2* RNAi data revealed differentially expressed genes with known roles in mechanosensory functions, such as *loxhd-1*, *cdh23*, and *myo7a*. Mutations in these genes can cause a loss of mechanosensation/transduction. For example, a recent study has demonstrated that *loxhd-1* mutation in the inner hair cell does not affect the structural integrity of the hair cell bundle but rather prevents the activation of MET (mechanoelectrical transducer) channels, thereby contributing to progressive hearing loss in mice (Trouillet et al., 2021). Other interesting candidates included a planarian homolog of Ephrin receptors, *Smed-Eph1* (*Eph1*), which was also expressed in *soxB1–2*^+^*/pou4-2*^+^ neurons and was downstream of these two transcription factors. Little is known about the role of Ephrin signaling in planarian regeneration. We discovered that *Eph1* is required for patterning of mechanosensory neurons in *S. mediterranea* (McCubbin, 2022; Warner, 2024), which led us to examine in detail how Ephrin signaling genes contribute to neural patterning in planarians (unpublished observations). Thus, as we demonstrated with *soxB1–2* (Ross et al., 2024; Ross et al., 2018), planarians are also useful to analyze *pou4-2*-regulated genes and their roles in cell differentiation or mechanosensation. It will be important to perform a comparative analysis of Pou4-regulated genes gleaned from other animals, like sea anemones or vertebrates like birds and fish, which can regenerate hair cells or the lateral line, respectively (Chen et al., 2019; Tourniere et al., 2020; Zhao et al., 2020). However, we found that our RNA-seq experimental design was limited in detecting *pou4-2*-regulated transcripts due to the limitations of systemic RNAi and collecting RNA from how animals. Before leveraging existing transcriptomic data on Pou4 homologs or hair cell or lateral line regeneration (Jiang et al., 2014; Ku et al., 2014; Tourniere et al., 2020) for orthologous comparisons in other species to test to what extent the Pou4 gene regulatory kernel is conserved among these widely divergent animals, we have designed new experiments to enrich for *pou4-2*-expressing planarian tissues and have performed RNA-seq experiments producing a larger differentially expressed gene set (unpublished). In addition, ATAC-seq experiments could be performed to examine how *pou4-2* activity affects chromatin architecture. These future genomic experiments should build upon this work and improve the resolution of the *pou4-2* gene regulatory network implicated in sensory neuron regeneration.

*pou4-2* function could encompass additional roles other than the ones identified in this study. In addition to the *pkd1L-2*^+^ and *hmcn-1-L*^+^ populations present in the rheosensory organ, there is a population of *pou4-2*^+^ cells in the ventral nerve cords and cephalic ganglia, which are not regulated by SoxB1–2 (Figure 1A). Although the ventral nerve cords are populated by *npp-3*^+^ cells, Pou4-2 activity does not regulate *npp-3* expression in this region as it does in the rheosensory organ (Figure 5B), and it has not been confirmed whether any cells in the ventral nerve cords are *pou4-2*^+^/*npp-3*^+^ co-expressing cells. This work did not elucidate the function of this pou4-2+ central nervous system cell population, but it may be possible to mine new scRNA-seq datasets to uncover transcription factors, such as in King et al. (2024), to predict the identities of *pou4-2*^+^ cells negative for expression of sensory neuron markers like *pkd1L-2* and *hmcn-1-L*. It was surprising to find that the relationship between *pou4-2* and *atonal* is not conserved in planarians, but the expression patterns of planarian *atonal* genes indicated that they represent completely different cell populations from *pou4-2*-regulated mechanosensory neurons. Although many *pou4-2*-regulated genes are expressed in additional tissues separate from the rheosensory organ, Pou4-2 predominantly appears to regulate gene expression in areas enriched with mechanosensory cells, consistent with observations in *pou4-2(RNAi)* planarians in other studies (Elliott, 2016; King et al., 2024).

### *Smed-pou4-2* is required for regeneration of mechanosensory function

RNAi of *pou4-2* eliminated the expression of terminally differentiated *pkd1L-2* and *hmcn-1-L* mechanosensory neurons in intact animals, whereas *pou4-2*(*RNAi*) regenerates had a low, dispersed expression of these genes at the head tips and peripheral ciliated stripes (Figure 4B). We speculate that following injury, Pou4-2^+^ post-mitotic progenitors or committed cells can still differentiate into sensory neurons in response to injury and polarity cues. This is consistent with previous observations in animals treated with hydroxyurea (HU) to block stem cell progression through S-phase. In these experiments, *Smed-APC-1(RNAi)* and *Smed-ptc(RNAi)* HU-treated animals were still able to regenerate neurons (Evans et al., 2011). It is also consistent with the hypothesis that many planarian stem cells are already specialized (Raz et al., 2021) and may have been unaffected by our RNAi treatment scheme. Nevertheless, both *pou4-2*(*RNAi*) intact animals and regenerates exhibited a significant reduction in mechanosensation (i.e., rheosensation and mechanosensation) relative to controls (Figure 6D–E). Moreover, the late re-expression of *pou4-2* during regeneration and the faster depletion rate of *pou4-2*^+^ cells that were negative for markers of terminal differentiation, *pkd1L-2h and hmcn-1-L*, compared to loss of *pou4-2*^+^ cells positive for the same terminal markers in irradiated planarians (Supplementary Figure S3) points to the *pou4-2*^+^ cells as progenitors responsible for generating terminally differentiated *pkd1L-2*^+^ and *hmcn-1-L*^+^ cells.

*pou4-2*^+^ cells in the dorsal head tip and peripheral and dorsal ciliated stripes (the planarian rheosensory organ) are downstream of SoxB1–2 activity, and RNAi and WISH experiments demonstrated that *pou4-2* expression is necessary for maintaining the functional properties of mechanosensory neurons (Figure 2D–E). However, the planarian rheosensory organ is composed of both ciliated mechanosensory neuronal and epidermal populations. Since *soxB1–2* is a pioneer transcription factor of the ectodermal lineage, RNAi of *soxB1–2* resulted in the loss of both ciliated sensory neuronal and epidermal populations in the rheosensory organ of planarian; thus, the dorsal ciliated stripe observed in acetylated tubulin labeling disappeared entirely (Ross et al., 2018). In contrast, in *pou4-2*(*RNAi*) animals, only mechanosensory neuronal populations were removed from the dorsal ciliated stripe, but modest labeling of ciliated epidermal populations persisted in the region (Figure 6A–B). Altogether, it can be concluded that *pou4-2*^+^ specifically marks subsets of planarian mechanosensory neuron populations and does not have a role in the regulation of the epidermal cells of the rheosensory organ, unlike SoxB1–2, which plays an earlier and broader regulatory role.

### Concluding Remarks

Despite molecular evidence indicating planarians possess ciliated mechanoreceptors sharing homology with mechanoreceptor function and development in other organisms, we have yet to resolve the cellular morphologies of the collection of cells comprising the ciliated stripes. Although it remains uncertain whether ciliated mechanoreceptors are products of convergent evolution or share a common cellular ancestry (Manley and Ladher, 2008), the role of Pou4 appears to represent a critical component of an adaptable gene regulatory network that has been co-opted to manufacture mechanoreceptors in distinct cell types. Secondly, we have yet to fully explore how *pou4*^+^ progenitors are born in response to local cues. Studies have implicated Notch signaling as the likely culprit (Elliott, 2016), and recent studies in *Schmidtea mediterranea* have elegantly demonstrated that Notch signaling plays a role in patterning neurons and glial cells (Scimone et al., 2025). This work demonstrates that *pou4-2* plays a key role in ciliated mechanoreceptor maintenance, regeneration, and function in *S. mediterranea*. Future studies will help to identify additional target *pou4-2* genes and further contribute to understanding how Pou4-2 regulates chromatin to facilitate the regeneration of mechanosensing cells in planarians.

## EXPERIMENTAL METHODS

### Planarian culture

Asexual clonal line CIW4 of *S. mediterranea* were maintained in 1x Montjuïc salts (1.6 mM NaCl, 1.0 mM CaCl_2_, 1.0 mM MgSO_4_, 0.1 mM MgCl_2_, 0.1 mM KCl, and 1.2 mM NaHCO_3_) in the dark at 20°C and fed weekly with pureed calf liver (Merryman et al., 2018). Planarians 3–5 mm in length were starved one week before experimentation unless specified otherwise.

### Gene identification and cloning

Sequences were obtained from an EST library (Zayas et al., 2005), cloned using gene-specific primers, or synthesized as eBlocks (IDT) and inserted into pPR-T4P (Liu et al., 2013) or pJC53.2 (Collins et al., 2010) vectors through ligation-independent cloning. Supplementary File S5 lists the primers used in cloning, eBlock sequences, and the EST clone accession numbers.

### scRNA sequencing data analysis

To infer the gene expression profiles of *pou4-2*^+^ cells, we analyzed publicly available scRNA-seq data from *S. mediterranea* [GSE111764] (Fincher et al., 2018). From the whole-body data (50562 cells) and the brain data (7766 cells), we extracted putative neuronal cells, which we define as cells expressing at least one of the following transcripts: *synapsin* (dd_Smed_v4_3135_0_1), *synaptotagmin* (dd_Smed_v4_4222_0_1, dd_Smed_v4_6730_0_1, dd_Smed_v4_6920_0_1), and *synaptosome associated protein 25* (dd_Smed_v4_13079_0_1, dd_Smed_v4_13255_0_1, or dd_Smed_v4_3977_0_1), which yielded a total of 20,557 cells. From these, we isolated a subset of 1427 neuronal cells expressing *soxB1–2* (dd_Smed_v4_8104_0_1). Expression data were scaled and transformed using standard functions of the R library Seurat (Satija et al., 2015). Visualization was done using UMAP dimensional reduction into two dimensions. Supervised community detection (clustering) was done using the built-in Leiden clustering algorithm (Traag et al., 2019). Since the UMAP projection suggested communities of disparate size, we used the Leiden algorithm in two steps – once with a smaller resolution to capture the large-scale structure (10 clusters) and then selectively with a higher resolution to resolve smaller communities, for a total of 19 distinct clusters. We characterized each cluster by finding differentially expressed genes (markers) using the bootstrap method of Pollard and van der Laan (Pollard and Laan, 2005) to calculate the *z*-score for every gene between cells in each cluster relative to cells outside the cluster (Supplementary File S1). The *z*-scores are then assessed for significance using the empirical Bayes method of Efron (Efron, 2008). The result is a posterior error probability *lfdr* assigned to each gene.

### RNA interference (RNAi)

Bacterially-expressed dsRNA was prepared by growing HT115 cells containing genes of interest ligated into pPR-T4P (Liu et al., 2013) or pJC.53.2 (Collins et al., 2010) vectors. Briefly, cultures grown overnight in LB with appropriate antibiotics were diluted in 40 mL 2xYT and incubated at 37°C and shaking at 225 rpm. Once cultures reached 0.6–0.8 OD600, 1 mM IPTG was added to induce dsRNA synthesis, and incubation continued for 2 hours. Cultures were then pelleted at 3,000 × g for 10 minutes at 4°C, resuspended in 8 mL LB, and aliquoted into 8 microcentrifuge tubes. Resuspended cultures were pelleted at 11,000 × g for 5 minutes at 4°C, aspirated, and stored at −80°C. For RNAi feeding, bacterial pellets were mixed with liver puree as previously described (Gurley et al., 2008) and planarians were fed 8 times over 4 weeks. *gfp* dsRNA was used as the control for all RNAi experiments. Intact animals were fixed 10 days after the 8^th^ feed, and regenerated animals were pre-pharyngeally amputated one day after the 8^th^ feed and fixed after 10 days of regeneration. Alternatively, RNAi feedings for the behavioral assays were performed using *in vitro* transcribed dsRNA mixed with pureed liver and agarose, as described in (Ross et al., 2018). The total numbers of animals for RNAi experiments are summarized in Supplementary File S6.

### *In situ* hybridization

Riboprobes were synthesized using an *in vitro* transcription reaction from DNA templates with digoxigenin or fluorescein-labeled NTPs, and whole-mount *in situ* hybridizations were performed as previously described (King and Newmark, 2013) in an InsituPro automated liquid handling robot (CEM Corporation, Matthews, NC). Briefly, samples were incubated with anti-Digoxigenin-AP (1:2000, Roche) for chromogenic detection, and the signals were subsequently developed with NBT/BCIP in AP buffer. For double fluorescent in situ hybridizations (dFISH), samples were incubated for 16 hours at 4°C with anti-DIG-AP and anti-FITC-POD (1:250, Roche). Peroxidase-conjugates were detected with tyramide signal amplification (TSA) as outlined previously in (Brown and Pearson, 2015), and Fast Blue development was utilized for AP-driven reaction detection (Lauter et al., 2011).

### RNA sequencing

Three biological replicates were obtained, each consisting of four worms of approximately four mm length at the start of the experiment, that were starved for one week prior to the start of RNAi feeding. Worms were fed bacterially expressed dsRNA three times on days 0, 3, and 7, and RNA was extracted and purified on day 12 (Allen et al., 2021). 500 ng of total RNA was used for the RNA-seq library preparation and sequencing at MedGenome, Inc. (Foster City, CA). The Poly-A-containing mRNA molecules were purified using poly-T oligo attached magnetic beads, and then mRNA was converted to cDNA using Illumina TruSeq stranded mRNA kit (20020595) according to the manufacturer’s protocol. Libraries were sequenced for 100 cycles to a depth of 30 million paired reads using Illumina NovaSeq 6000 (Illumina, San Diego, CA). The following quality control steps were performed on the fastq files: Base quality score distribution, Sequence quality score distribution, Average base content per read, GC distribution in the reads, distribution of over-represented sequences, and adapter trimming. Based on the quality report of fastq files, sequences were trimmed wherever necessary to retain only high-quality sequences for further analysis. In addition, the low-quality sequence reads are excluded from the analysis. Data quality check was performed using FastQC (v0.11.8). The adapter trimming was performed using the fastq-mcf program (v1.05) and cutadapt (v2.5) (Martin, 2011). Transcriptome alignment was performed using RSEM (version RSEM v1.3.1) (Li and Dewey, 2011) against the dd_Smed_v6 transcriptome (Rozanski et al., 2019) to build Bowtie transcriptome indexes using *rsem-prepare-reference*, then used *rsem-calculate-expression* for aligning and expression calculation. Differential expression analysis was performed using DESeq2 (R Bioconductor package) (Anders and Huber, 2010) with default parameters, and then differentially reduced genes were defined as those having a fold-change of <1.4 and p-adjusted value < 0.1 (see Supplementary File S3). The RNA sequencing data have been deposited in NCBI under BioProject accession PRJNA1258257. Gene Ontology (GO) annotation and over-presentation analysis, the unique set of *pou4-2(RNAi)* downregulated transcripts was compared to the human proteome (BLASTX against the Swiss-Prot Homo sapiens proteome, cutoff e-value < 1e^−3^). Human UniProt IDs were used for enrichment analysis using Fisher’s Exact tests with FDR multiple test correction (FDR < 0.05) in http://geneontology.org/. GO results are reported in Supplementary File S4.

### Immunohistochemistry

Animals were sacrificed in ice-cold 2% HCl for 30 seconds, followed by incubation in Carnoy’s fixative (6 parts ethanol, 3 parts CHCl3, 1 part glacial acetic acid) for 2 hours at 4°C (Forsthoefel et al., 2018), followed by a dehydration step with 100% methanol for 1 hour at 4°C. The animals were bleached overnight under a lamp with 6% H_2_O_2_ in methanol and then rehydrated in 75%, 50%, and 25% methanol-PBSTx, followed by two 5-minute PBSTx washes. PBSTb (1% BSA in PBSTx) was used for blocking at room temperature for 2 hours. Primary antibody labeling was carried out with mouse anti-Acetylated Tubulin (Sigma-Aldrich, St. Louis, MO) diluted in PBSTb (1:1000) overnight at 4°C. Six 1-hour PBSTx washes followed by 1 hour of PBSTb blocking were performed before anti-mouse-HRP (1:1000, Cell Signaling) incubation overnight at 4°C. After six 1-hour PBSTx washes, acetylated tubulin was detected through TSA development as described in (Brown et al 2015) with the following exceptions: no 4-IPBA or dextran sulfate was added to TSA reaction buffer, Cy3-tyramide was diluted 1:250, and development took place for a total of 20 minutes.

### Mechanosensation (vibration) assay

Analysis of the planarian’s ability to detect a vibration stimulus was conducted essentially as described in (Ross et al., 2024). Briefly, groups of five control *gfp*(*RNAi*) or *pou4-2*(*RNAi*) planarians were added to a 100 × 15 mm petri dish containing 40 ml of 1x Montjuïc salts that was placed inside a dish lid that was mounted to a cold LED lighted board using clear silicone paste and observed until gliding normally. Then, an Arduino-controlled arm delivered five taps at a rate of one tap every 75 msec to the side of the dish. Experimental runs were recorded on a Basler Ace 2 Pro ac1440–220uc camera connected to a PC running Basler’s pylon Viewer 64-bit version 6.3.0 software at a frame rate of 10 frames/sec and a frame size of 1440 × 1080 pixels. The video frames were analyzed in Fiji (ImageJ2 version 2.9.0) (Schindelin et al., 2012), using the line tool to measure the longest pre-stimulus gliding length and the length of the worms following the stimulus. The percent change in length was calculated as [(Length_Prestimulus_ – Length_Poststimulus_)/Length_Prestimulus_] × 100. Statistical analysis and graph generation were performed in GraphPad Prism 9 (GraphPad Software, Boston, MA). One-way ANOVA analyses were performed and corrected using Dunnett’s correction. All means were compared to the control group, and statistical significance was accepted at values of *p* < 0.01.

### X-ray irradiation

One-week starved animals measuring 3–4 mm in length were exposed to 100 Gray (Gy) of X-ray irradiation (130 kV, 5 mA, 8.4 Gy/min) for approximately 12 minutes using a CellRad X-ray Irradiator System (Precision X-Ray, Madison, CT).

### Imaging

The *in situ* hybridization images were acquired using a Leica DFC450 camera mounted on a Leica M205 stereomicroscope. Animals processed for fluorescent *in situ* hybridization and immunohistochemistry were mounted in Vectashield diluted 1:1 in 80% glycerol. Fluorescent images were acquired using a Zeiss AxioZoom equipped with an Apotome using Zen Pro version. High-magnification acetylated tubulin images were acquired using a Zeiss Axio Observer Inverted Microscope equipped with an AV4 Mod Apotome using AxioVision v4.6 (Carl Zeiss Microscopy, LLC, White Plains, NY).

### Cell counting and quantification

For co-labeling experiments between *pou4-2*-regulated genes with *pkd1L-2* or *hmcn-1-L*, maximum intensity projections of stacked fluorescent images were acquired with a depth of 12 μm. For *pou4-2* co-labeling with *pkd1L-2* and *hmcn-1-L* in irradiated planarians, maximum intensity projections of stacked fluorescent images were acquired with a depth of 24 μm. The regions quantified spanned the width of the sensory neuron expression pattern in the head tip and 500 μm anterior to the head tip along the length of the dorsal ciliated stripe. Cells were manually counted using Zen Lite v3.3. Cell quantification was represented as the number of positive cells per mm^2^, and graphs were made using GraphPad Prism (GraphPad Software, Boston, MA). Three to six biological replicates per group were used to quantify co-labeling. Cell quantification details and the total number of cells counted for Figure 4A and Supplementary Figure S3 are summarized in Supplementary Files S7 and S8, respectively.

## Supplementary Material

Supplement 1

Supplement 2

Supplement 3

Supplement 4

Supplement 5

Supplement 6

Supplement 7

Supplement 8

1 **Supplementary Figure 1.** (A) Schematic of RNAi treatment time course used prior to whole-mount in situ hybridization analysis for uninjured and regenerating animals, respectively. Planarians were fed twice per week for 4 weeks, amputated pre-pharyngeally one day after the last feeding, and allowed to regenerate for 10 days before fixation. (B) *pou4-2* RNAi caused a reduction in *soxB1–2* expression in the dorsal ciliated stripe (dcs). Anterior is to the top. Scale bars = 200 μm; n ≥ 3 worms tested, with all samples displaying similar expression patterns.
**Supplementary Figure 2.** Reciprocal RNAi/WISH analysis of *pou4-2* and *atonal* genes. (A) showed no changes in expression compared to controls. Scale bars = 200 μm (whole worms) and 100 μm (head region); n ≥ 3 worms tested with all samples displaying similar expression patterns (see Supplementary File S6).
**Supplementary Figure 3: *pou4-2* is expressed in irradiation-sensitive cells.** (A) Double fluorescent whole-mount in situ hybridization revealed *pou4-2* co-expression with *pkd1L-2* or *hmcn-1-L*. White boxed cells zoomed in within insets show high *pou4-2* and terminal marker expression and are displayed at higher magnification. White arrowheads point to examples where terminal marker gene expression is much brighter than *pou4-2* expression. White arrows mark *pou4-2*^+^ cells with low expression of the terminal marker genes. Scale bar = 100 μm. (B) timecourse X-ray exposure experiment to examine *pou4-2* expression in presumptive progenitor cells. Double-FISH detection of *pou4-2* with a *pkd1L-2* and *hmcn-1-L* riboprobe mix was severely reduced after 5.5 days post-irradiation (dpi). Red arrows highlight cells expressing *pou4-2* only. Scale bars = 200 μm. (B-C) Plots of measurements to track the number of *pou4-2*^+^/*pkd1L-2*^−^ *hmcn-1-L*^−^ cells (B) or *pou4-2*^+^*/pkd1L-2*^+^ and *pou4-2*^+^*/hmcn-1-L*^+^ (C) per mm^2^ over days post-irradiation. (D) Spatiotemporal expression of *piwi-1* after 100 Gy irradiation along with early-stage epidermal progenitor prog and late-stage epidermal progenitor *agat-1*. The WISH analysis revealed that *pou4-2* shares the same spatiotemporal expression pattern as *agat-1*.
