# Supplementary material for: Smed-pou4-2 regulates mechanosensory neuron regeneration and function in planarians
Source: bioRxiv. 2025 Sep 11:2025.05.15.654132. Originally published 2025 May 16. Preprint. [Version 2] doi: 10.1101/2025.05.15.654132 (PMC12132226; doi:10.1101/2025.05.15.654132)

bioRxiv preprint doi: https://doi.org/10.1101/2025.05.15.654132; this version posted September 11, 2025. The copyright holder for this preprint (which was not certified by peer review) is the author/funder, who has granted bioRxiv a license to display the preprint in perpetuity. It is made available under aCC-BY 4.0 International license.

SUPPLEMENTARY FIGURE 1

SMED30002016 expression determined by RNA-seq during embryonic development stages in *Schmidtea mediterranea*.

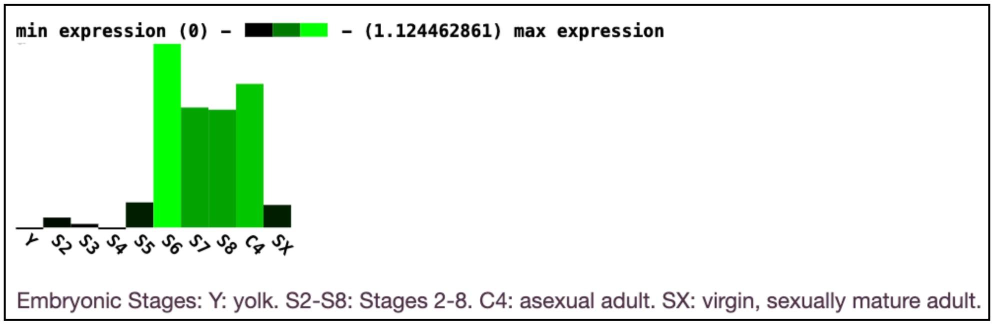

Adapted from: https://planosphere.stowers.org/feature/Schmitea/mediterranea-sexual/transcript/SMED30002016.

bioRxiv preprint doi: https://doi.org/10.1101/2025.05.15.654132; this version posted September 11, 2025. The copyright holder for this preprint (which was not certified by peer review) is the author/funder, who has granted bioRxiv a license to display the preprint in perpetuity. It is made available under aCC-BY 4.0 International license.

SUPPLEMENTARY FIGURE 2

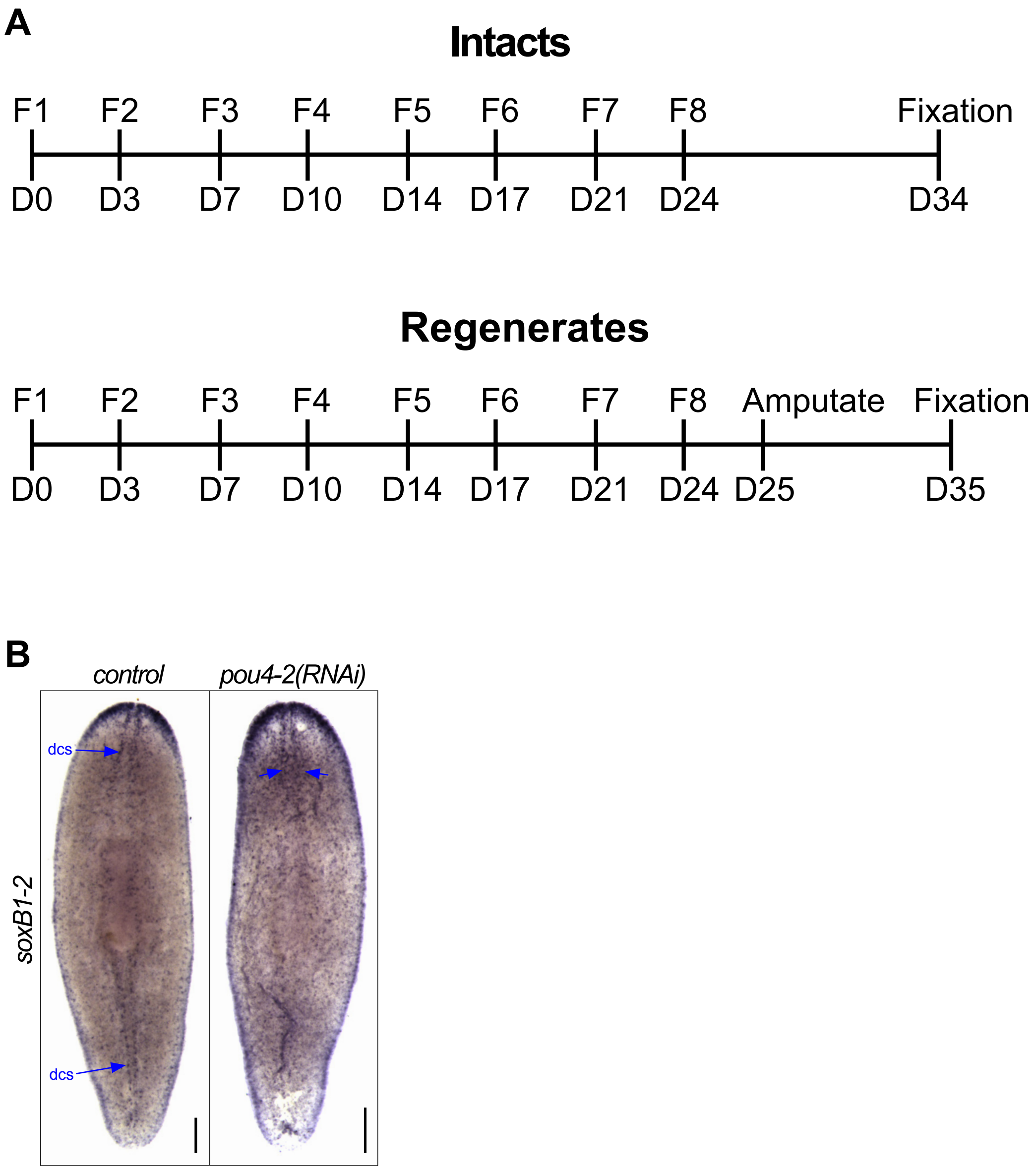

bioRxiv preprint doi: https://doi.org/10.1101/2025.05.15.654132; this version posted September 11, 2025. The copyright holder for this preprint (which was not certified by peer review) is the author/funder, who has granted bioRxiv a license to display the preprint in perpetuity. It is made available under aCC-BY 4.0 International license.

SUPPLEMENTARY FIGURE 3

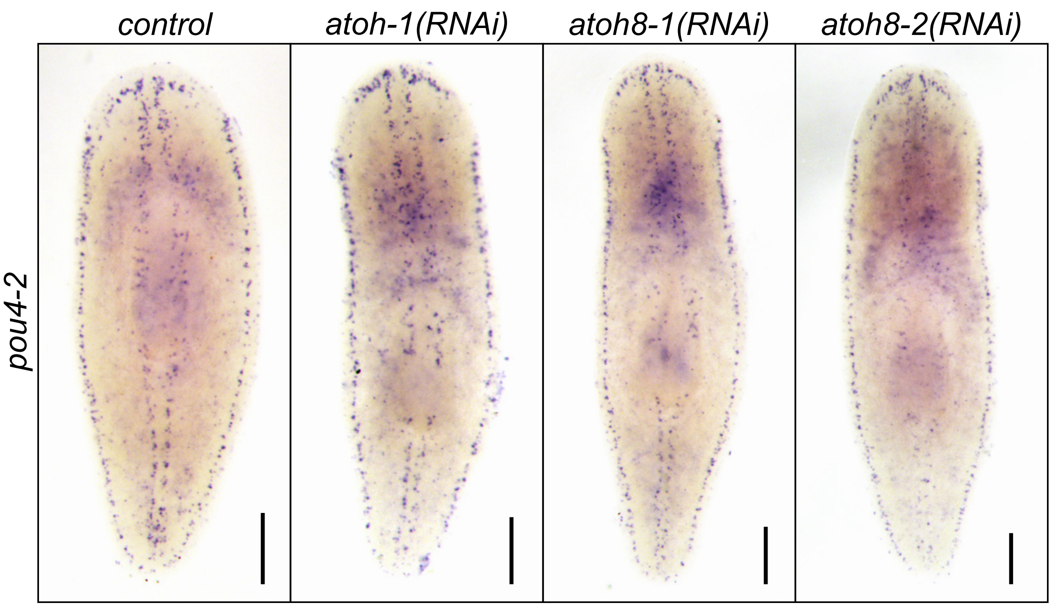

bioRxiv preprint doi: https://doi.org/10.1101/2025.05.15.654132; this version posted September 11, 2025. The copyright holder for this preprint (which was not certified by peer review) is the author/funder, who has granted bioRxiv a license to display the preprint in perpetuity. It is made available under aCC-BY 4.0 International license.

SUPPLEMENTARY FIGURE 4

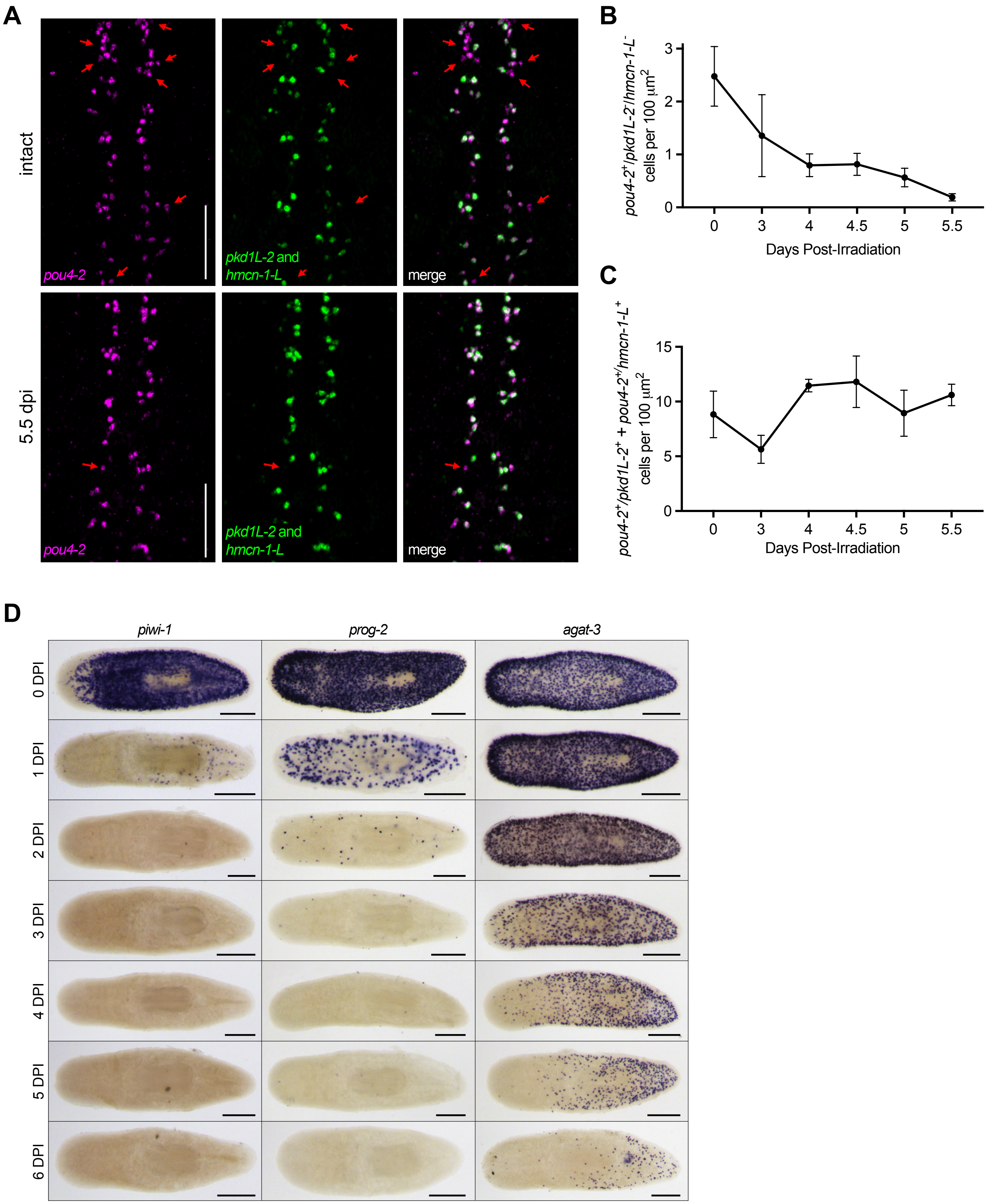

Supplement: 9 — Supplementary Figure 1: Expression of Smed-pou4-2 during development of S. mediterranea. BLAST in Planosphere cross-referenced dd_Smed_v6_30562_0_1 to SMED30002016 (E-value = 0). RNA sequencing data from Davies et al. (2017) show that Smed-pou4-2 expression peaks during stages 5-6, coinciding with organogenesis and axial patterning. Expression levels are comparable to those in mature asexual worms. Supplementary Figure 2: Schematic of RNAi treatment and reciprocal expression analysis. (A) Timeline of RNAi feeding and fixation for WISH in uninjured and regenerating animals. Planarians were fed twice per week for 4 weeks, amputated pre-pharyngeally one day after the final feeding, and fixed 10 days post-amputation for WISH analyses. (B) pou4-2 RNAi leads to reduced soxB1-2 expression in the dorsal ciliated stripe (dcs). Anterior is to the top. Scale bars = 200 μm; n ≥ 3 worms tested, with all samples displaying similar expression patterns. Supplementary Figure 3: Knockdown of atonal genes does not alter pou4-2 expression in regenerating animals. Scale bars = 200 μm; n ≥ 3 worms per group (see Supplementary File S6). Supplementary Figure 4: Irradiation reveals that pou4-2+ cells include progenitors. (A) Time-course analysis following 100 Gy X-ray exposure shows progressive loss of pou4-2+/terminal marker− presumptive progenitor cells. Double FISH with a combined pkd1L-2/hmcn-1-L riboprobe reveals reduced labeling by 5.5 days post-irradiation (dpi). Red arrows mark pou4-2+ cells lacking terminal marker expression. Scale bars = 200 μm. (B-C) Quantification of pou4-2+/pkd1L-2− hmcn-1-L− cells (B) or pou4-2+/pkd1L-2+ plus pou4-2+/hmcn-1-L+ (C) per mm2 dpi. (D) WISH analysis of piwi-1, prog-1, and agat-1 post-irradiation reveals that the pou4-2+/terminal marker− putative progenitors share a spatiotemporal depletion pattern with late progenitor marker agat-1+ cells. [file NIHPP2025.05.15.654132v2-supplement-9.pdf]
