# Supplementary material for: Smed-pou4-2 regulates mechanosensory neuron regeneration and function in planarians
Source: bioRxiv. 2025 May 16:2025.05.15.654132. Preprint. [Version 1] doi: 10.1101/2025.05.15.654132 (PMC12132226; doi:10.1101/2025.05.15.654132)

bioRxiv preprint doi: https://doi.org/10.1101/2025.05.15.654132; this version posted May 16, 2025. The copyright holder for this preprint (which was not certified by peer review) is the author/funder, who has granted bioRxiv a license to display the preprint in perpetuity. It is made available under aCC-BY 4.0 International license.

SUPPLEMENTARY FIGURE 1

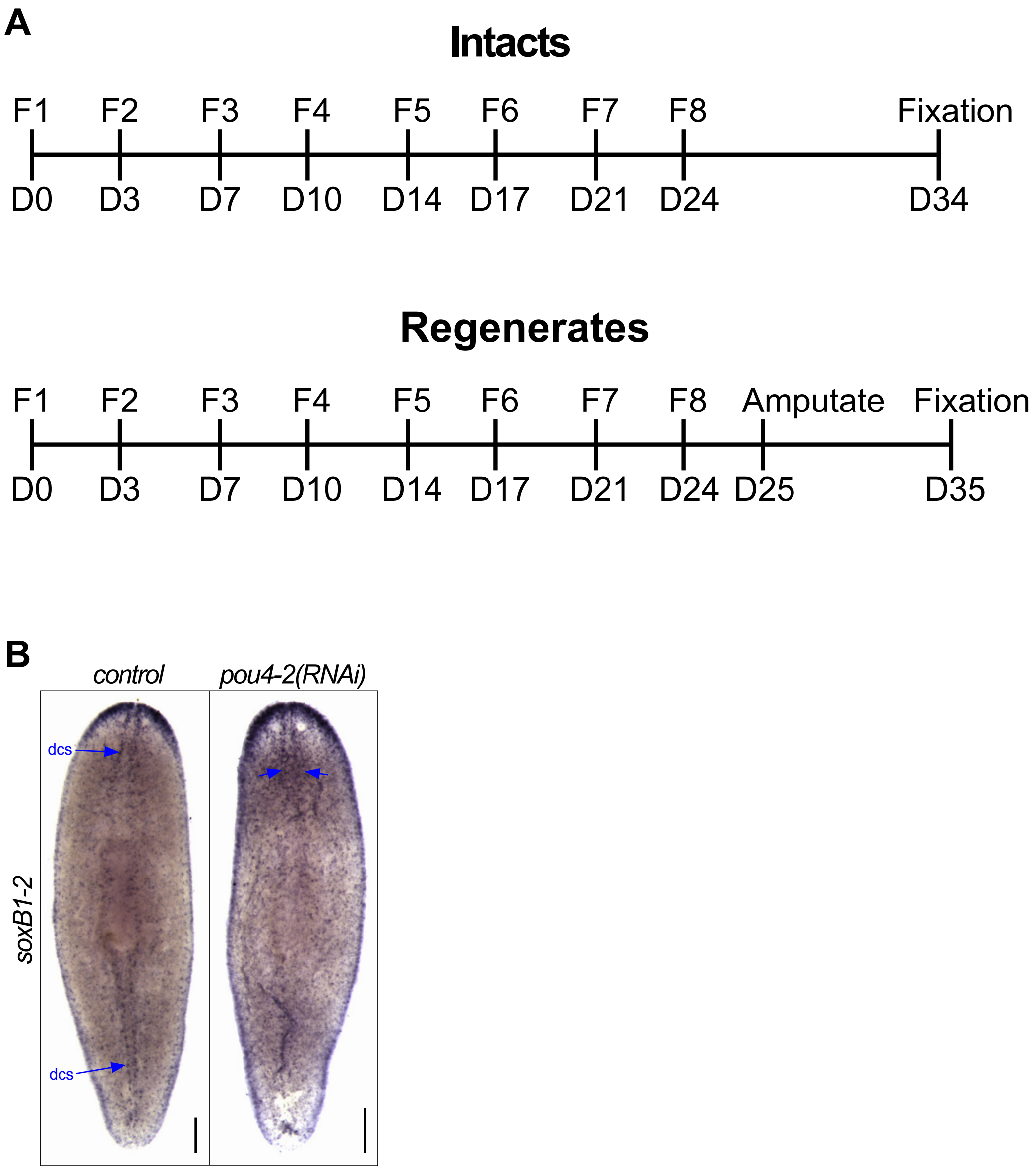

bioRxiv preprint doi: https://doi.org/10.1101/2025.05.15.654132; this version posted May 16, 2025. The copyright holder for this preprint (which was not certified by peer review) is the author/funder, who has granted bioRxiv a license to display the preprint in perpetuity. It is made available under aCC-BY 4.0 International license.

SUPPLEMENTARY FIGURE 2

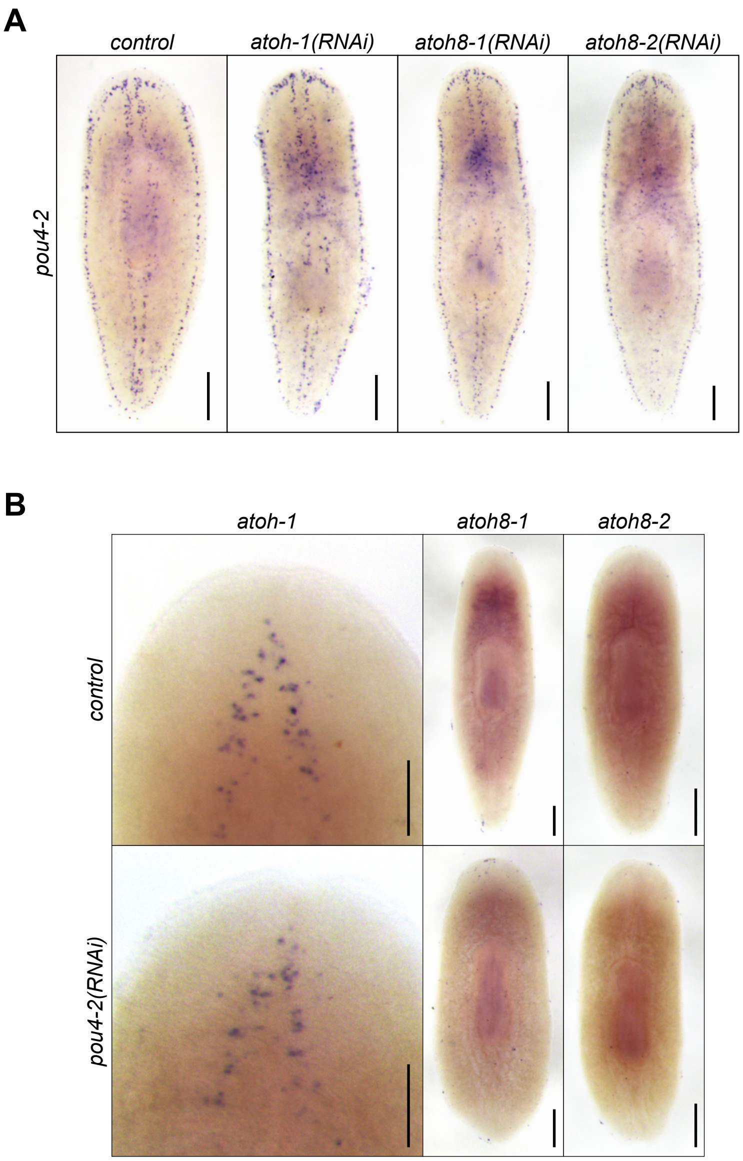

bioRxiv preprint doi: https://doi.org/10.1101/2025.05.15.654132; this version posted May 16, 2025. The copyright holder for this preprint (which was not certified by peer review) is the author/funder, who has granted bioRxiv a license to display the preprint in perpetuity. It is made available under aCC-BY 4.0 International license.

SUPPLEMENTARY FIGURE 3

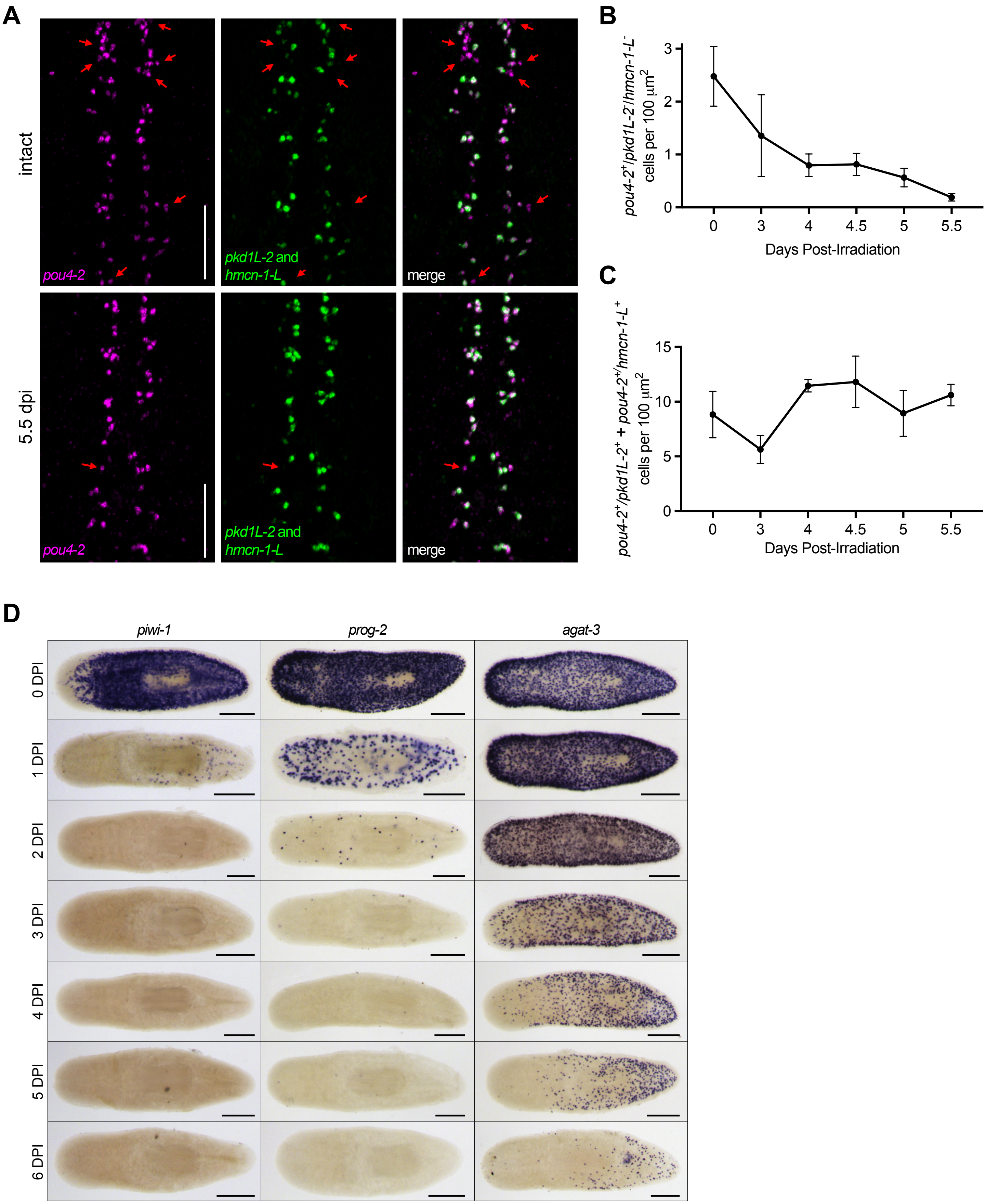

Supplement: Supplementary file 1 [file NIHPP2025.05.15.654132V1-supplement-1.pdf]
